# Supplementary material for: Temperature dependence laser-assist scattering and its impact on efficiency of PEMFC
Source: PLoS One. 2026 Jan 9;21(1):e0336352. doi: 10.1371/journal.pone.0336352 (PMC12788626; doi:10.1371/journal.pone.0336352)
Supplement: Attachment — Submitted filename: PLOS ONE-review-report1.pdf [file pone.0336352.s002.pdf]

Response to Reviewers

First of all, the authors would like to thank the editorial team for providing smooth communication throughout the review process. We also extend our sincere gratitude to all reviewers for their valuable suggestions and comments, which have significantly helped improve the manuscript for better clarity and readability. All corrections, edits, and modifications have been incorporated and highlighted in the revised manuscript to support easy re-evaluation. Additionally, point-by-point responses to the reviewers' comments are provided below.

PONE-D-25-41737

Temperature Dependence Laser-Assist Scattering and Its Impact on Efficiency on with PEMFC

Editorial comment

Please include the following items when submitting your revised manuscript:

• A rebuttal letter that responds to each point raised by the academic editor and reviewer(s). You should upload this letter as a separate file labeled 'Response to Reviewers'.

Respond to reviewer; We revised point wise the comment of reviewers and editorials team. The pointwise revisions reply to editorial team and reviewers are below with highlighted in manuscript.

• A marked-up copy of your manuscript that highlights changes made to the original version. You should upload this as a separate file labeled 'Revised Manuscript with Track Changes'.

Respond to reviewer; The comment is considered as manuscript is revised with highlighted in track change

• An unmarked version of your revised paper without tracked changes. You should upload this as a separate file labeled 'Manuscript'.

Respond to reviewer; This comment is also considered during revised submission

If you would like to make changes to your financial disclosure, please include your updated statement in your cover letter. Guidelines for resubmitting your figure files are available below the reviewer comments at the end of this letter.

Respond to reviewer; No, we don't change financial disclosure. In addition, I would like to know editorial team our work is funded but for research and research fund don't cover APC and we see our country are in group A for reserch4life list.

If applicable, we recommend that you deposit your laboratory protocols in protocols.io to enhance the reproducibility of your results. Protocols.io assigns your protocol its own identifier (DOI) so that it can be cited independently in the future. For instructions see: https://journals.plos.org/plosone/s/submission-guidelines#loc-laboratory-protocols. Additionally, PLOS ONE offers an option for publishing peer-reviewed Lab Protocol articles, which describe protocols hosted on protocols.io. Read more information on sharing protocols at https://plos.org/protocols?utm_medium=editorial-email&utm_source=authorletters&utm_campaign=protocols.

Respond to reviewer; Yes ,we created and deposits the laboratory protocol. And uploaded;

https://dx.doi.org/10.17504/protocols.io.e6nvw436zlmk/v1

When submitting your revision, we need you to address these additional requirements.

1. Please ensure that your manuscript meets PLOS ONE's style requirements, including those for file naming. The PLOS ONE style templates can be found at

Respond to reviewer; We submit the revised-on PLOS ONE's style.

2. Please note that PLOS One has specific guidelines on code sharing for submissions in which author-generated code underpins the findings in the manuscript. In these cases, we expect all author-generated code to be made available without restrictions upon publication of the work. Please review our guidelines at https://journals.plos.org/plosone/s/materials-and-software-sharing#loc-sharing-code and ensure that your code is shared in a way that follows best practice and facilitates reproducibility and reuse.

Respond to reviewer; we try to share the code but unable to share so we are submitting the code with revised manuscript with file name Plos-one code.

3. We note that the grant information you provided in the 'Funding Information' and 'Financial Disclosure' sections do not match.

Respond to reviewer; Added a section as Funding Information and Financial Disclosure where we include grant information detial.

When you resubmit, please ensure that you provide the correct grant numbers for the awards you received for your study in the 'Funding Information' section.

4. Thank you for stating the following financial disclosure: "UGC-Nepal"

Respond to reviewer; We revised and added

Please state what role the funders took in the study. If the funders had no role, please state: "The funders had no role in study design, data collection and analysis, decision to publish, or preparation of the manuscript."

If this statement is not correct you must amend it as needed.

Please include this amended Role of Funder statement in your cover letter; we will change the online submission form on your behalf.

Respond to reviewer; The funders had no role regarding to manuscript preparation and publication.

5. **Thank you for stating the following in your Competing Interests section:**

"Authors has no competing interest"

Please complete your Competing Interests on the online submission form to state any Competing Interests. If you have no competing interests, please state "The authors have declared that no competing interests exist.", as detailed online in our guide for authors at http://journals.plos.org/plosone/s/submit-now

Respond to reviewer; We revised and edited as 'The authors have declared that no competing interests exist" in competing interest

This information should be included in your cover letter; we will change the online submission form on your behalf.

6. Please provide a complete Data Availability Statement in the submission form, ensuring you include all necessary access information or a reason for why you are unable to make your data freely accessible. If your research concerns only data provided within your submission, please write "All data are in the manuscript and/or supporting information files" as your Data Availability Statement.

Respond to reviewer; Data and code should be available on request by interested researcher. This is because code is very import and it's may be misused if interested anyone can contact corresponding authors with mutual understanding**.**

7. Please include your full ethics statement in the 'Methods' section of your manuscript file. In your statement, please include the full name of the IRB or ethics committee who approved or waived your study, as well as whether or not you obtained informed written or verbal consent. If consent was waived for your study, please include this information in your statement as well.

Respond to reviewer; Ethical approvals are not needed this work because it is computational work where we self-design the theoretical model. The experimental part doesn't include any human/animals trail,

8. If the reviewer comments include a recommendation to cite specific previously published works, please review and evaluate these publications to determine whether they are relevant and should be cited. There is no requirement to cite these works unless the editor has indicated otherwise.

Respond to reviewer; Thank for suggestion we will take cure of it. In addition, reviewer don't have suggest any citation or literature.

Reviewers' comments:

1. Is the manuscript technically sound, and do the data support the conclusions?

The manuscript must describe a technically sound piece of scientific research with data that supports the conclusions. Experiments must have been conducted rigorously, with appropriate controls, replication, and sample sizes. The conclusions must be drawn appropriately based on the data presented.

Reviewer #1: Yes

Reviewer #2: Partly

Respond to reviewer; the experiment is replication because re revised some part with highlighted so that reader can replicate it. In addition, for sample size we added plot with standard deviation which fulfil the limitation of reviwer comment sample size.

2. Has the statistical analysis been performed appropriately and rigorously?

Reviewer #1: Yes

Reviewer #2: No

Respond to reviewer; also added some addition statistical analysis like uncertainty in measurement and highlighted in revised manuscript.

3. Have the authors made all data underlying the findings in their manuscript fully available?

The PLOS Data policy requires authors to make all data underlying the findings described in their manuscript fully available without restriction, with rare exception (please refer to the Data Availability Statement in the manuscript PDF file). The data should be provided as part of the manuscript or its supporting information, or deposited to a public repository. For example, in addition to summary statistics, the data points behind means, medians and variance measures should be available. If there are restrictions on publicly sharing data—e.g. participant privacy or use of data from a third party—those must be specified.

Reviewer #1: Yes

Reviewer #2: Yes

Respond to reviewer; Yes we agreed the reviewers and also considered the editorial board comment of this comment.

4. Is the manuscript presented in an intelligible fashion and written in standard English?

PLOS ONE does not copyedit accepted manuscripts, so the language in submitted articles must be clear, correct, and unambiguous. Any typographical or grammatical errors should be corrected at revision, so please note any specific errors here.

Reviewer #1: Yes

Reviewer #2: Yes

Respond to reviewer; We agree with reviwer and also goes throughout the manuscript to minimized such error.

5. Review Comments to the Author

Please use the space provided to explain your answers to the questions above. You may also include additional comments for the author, including concerns about dual publication, research ethics, or publication ethics. (Please upload your review as an attachment if it exceeds 20,000 characters)

Reviewer #1: The paper concludes that external temperature negatively affects PEMFC output due to increased particle resistance and entropy, emphasizing the need for precise thermal management to improve fuel cell efficiency. However， the experimental setup is too simple. It is recommended to upgrade the experimental conditions to ensure the authenticity of the experiment.

Respond to reviewer; we agree with reviwer that experimental is too simple but the interconnection of these simple experimental with desing theory is not easy. In addition, for validation we have no such experimental work which interlink the DCS with different parameters which we can extract form the googling till the present data. How ever we added some possible validation to the result.

Reviewer #2: This is a statistical review on a Research Article manuscript for PLOS ONE entitled "Temperature Dependence Laser-Assist Scattering and Its Impact on Efficiency on with PEMFC" (PONE-D-25-41737).

Respond to reviewer; we used statical review and added uncertainty on measurement including type a and type 2 error in revised manuscript.

This paper is primarily a theoretical modeling and descriptive experimental study rather than one based on inferential statistics. The authors develop a theoretical model and then conduct a single prototype experiment to observe a phenomenon. As a result, it lacks formal statistical components.

Respond to reviewer; Yes, we agree with reviewer and some statistical component is like standard error bar and uncertainty in measurement is added in revised manuscript.

The statistical methods used are limited to descriptive statistics.

Respond to reviewer; added in revised manuscript

There are no statistical experimental design considerations, no statistical modeling (or use of statistical models) and no inference (such as tests of statistical hypotheses).

Respond to reviewer; Actually, our hypothesis for the developed theory is temperatures increase DCS increase which we found in theoretical model while for this when we design experiment, we also found the voltage decrease as temperature increase which mean DCS increase which is theoretical validation with experimental. We try to verify the theoretical model to experimental

with nature not statical because we have not such advance facilities but we have verified with our resource. So statical modeling and statical experimental is neither possible no done in this work

The study uses a simple, non-replicated prototype design. A single PEMFC prototype was built and tested. There is no statistical sampling. The "sample" is a single, custom-fabricated device. The paper does not mention any replication of the experiment.

Respond to reviewer; Some revisions have been made to improve the replicability of the experimental model, and these changes are highlighted in the manuscript. After setting up the experiment, 1 mL of hydrogen was supplied to the system, and data were recorded five times to calculate the experimental uncertainty. When external factors such as heat were applied, the experiment was again repeated five times at the same temperature to obtain the corresponding uncertainty. By combining both uncertainties, we determined the overall measurement uncertainty. We hope this clarification appropriately addresses the reviewer's comment.

From the statistics standpoint, the paper fails to deliver on the following important rubric of PLOS ONE:

"3. Experiments, statistics, and other analyses are performed to a high technical standard and are described in sufficient detail."

Respond to reviewer; Details of the technical standards have been added, along with statistical analysis of the experimental data. Standard error bars have been included in the voltage–temperature plot, and a table of uncertainty measurements—based on four repetitions at each temperature—has been added and highlighted in the revised manuscript.

The above evaluation considers only statistical aspects of the paper as part of the requested statistical review of the manuscript. It does not reflect on the overall scientific merits of the presented research.

Respond to reviewer; We incorporated appropriate statistical analyses in the experiment, including plotting graphs with standard error bars and calculating measurement uncertainty by considering both Type A and Type B errors.

6. PLOS authors have the option to publish the peer review history of their article (what does this mean?). If published, this will include your full peer review and any attached files.

Respond to reviewer; Yes, we agree with peer review history of our article in published

If you choose "no", your identity will remain anonymous but your review may still be made public.

Respond to reviewer; We choose yes for peer review history for our article if published

Do you want your identity to be public for this peer review? For information about this choice, including consent withdrawal, please see our Privacy Policy.

Reviewer #1: No

Reviewer #2: No

Respond to reviewer; It is reviwer choice authors have no comment on it.

To ensure your figures meet our technical requirements, please review our figure guidelines: https://journals.plos.org/plosone/s/figures

You may also use PLOS's free figure tool, NAAS, to help you prepare publication quality figures: https://journals.plos.org/plosone/s/figures#loc-tools-for-figure-preparation.

NAAS will assess whether your figures meet our technical requirements by comparing each figure against our figure specifications.

Respond to reviewer; we reviwer figure guidelines of PLOS ONE's and revised the figure according to guidelines in revised manuscript.
